# Methylprednisolone as an adjunct to intravenous immunoglobulin in pediatric Guillain–Barré syndrome: a prospective comparative study

**DOI:** 10.1038/s41598-026-44160-w

**Published:** 2026-04-02

**Authors:** Abdel-Ghaffar Ismail Fayed, Abdelsattar Abdullah Elsayeh, Mohammad Ali Saeed Hassan

**Affiliations:** 1 Department of Neurology, Faculty of Medicine, Al-Azhar University, Cairo, Egypt; 2 Department of Pediatrics, Faculty of Medicine, Al-Azhar University, Cairo, Egypt

**Keywords:** Guillain–Barré syndrome, Pediatrics, Intravenous immunoglobulin, Methylprednisolone, Neuropathic pain, Functional recovery, Diseases, Health care, Medical research, Neurology, Neuroscience

## Abstract

Guillain–Barré syndrome (GBS) is the most common cause of acute flaccid paralysis in children. Intravenous immunoglobulin (IVIG) is considered the standard therapy; however, the potential benefit of adjunctive corticosteroids remains uncertain.This study aimed to explore whether the addition of short-course intravenous methylprednisolone to IVIG may accelerate recovery and alleviate neuropathic pain in pediatric patients with GBS. A prospective, comparative study was conducted at Al-Azhar University Hospitals, enrolling 28 pediatric patients with clinically and electrophysiologically confirmed GBS. Participants were randomized into two equal groups: Group A received IVIG alone (1 g/kg/day for two days), while Group B received IVIG combined with pulse-dose methylprednisolone (30 mg/kg/day for five days). Clinical outcomes including time to initial improvement, unaided ambulation, Hughes Disability Score, neuropathic pain, and adverse events were assessed over a six-month follow-up period. Baseline characteristics were comparable between groups. The combination therapy group demonstrated faster initial improvement (median 3 vs. 4.5 days), earlier independent ambulation (21 vs. 28 days), and modest improvement in Hughes score at three months (median 0 vs. 1, *p* = 0.016), though clinical significance is limited. Neuropathic pain resolved completely in the steroid group but persisted in 57.1% of the IVIG-only group (*p* = 0.002). By six months, nearly all patients achieved complete recovery. Adverse events in the combination group were limited to mild weight gain and increased appetite. Short-course methylprednisolone as an adjunct to IVIG may provide early benefits, particularly in pain resolution, without major safety concerns. These findings are preliminary and hypothesis-generating, requiring confirmation in larger multicenter trials. These findings highlight a feasible and safe strategy that could be particularly valuable in resource-limited healthcare settings, though confirmation in larger multicenter trials is warranted.

## Introduction

Guillain–Barré syndrome (GBS) is the most frequent cause of acute flaccid paralysis in children and is characterized by immune-mediated demyelination or axonal injury of peripheral nerves. Although relatively uncommon in the pediatric population, GBS carries a substantial risk of respiratory compromise, autonomic dysfunction, and long-term disability when diagnosis or treatment is delayed^1,2^.

Intravenous immunoglobulin (IVIG) remains the treatment of choice, supported by robust evidence demonstrating its ability to halt disease progression and facilitate neurological recovery^3,4^. In contrast, corticosteroids have shown limited efficacy when used as monotherapy^5,6^. Some pediatric studies have suggested that short-course methylprednisolone, when combined with IVIG, may promote faster recovery, though evidence remains limited and requires cautious interpretation in severe or rapidly progressive cases^7,8^.

Neuropathic pain is increasingly recognized as a significant burden in pediatric GBS, with major implications for mobility, rehabilitation, and quality of life^9^. Despite its clinical importance, few prospective studies have evaluated whether adjunctive corticosteroids can improve pain outcomes in this population.

Experimental and clinical evidence suggests that corticosteroids may modulate complement activation and cytokine-mediated injury, thereby reduce axonal damage and contribute to analgesic and functional benefits^10^. These mechanisms, together with the accessibility and low cost of corticosteroids compared with IVIG, make them a practical therapeutic adjunct-particularly in resource-constrained healthcare systems^11^.

The present prospective comparative study was designed to assess the clinical impact of combining intravenous methylprednisolone with IVIG in pediatric GBS. Specifically, we investigated whether this combination accelerates early recovery, facilitates ambulation, and reduces post-treatment neuropathic pain compared with IVIG alone in a tertiary Egyptian center.

## Materials and methods

### Study design and setting

This prospective, comparative study was conducted at the Department of Pediatrics, Al-Hussein Hospital, Faculty of Medicine, Al-Azhar University, Cairo, Egypt, over a 16-month period (November 2022 to February 2024).

### Participants

Twenty-eight consecutive pediatric patients (age range, e.g., 1–18 years), of both sexes, with a confirmed diagnosis of Guillain–Barré syndrome (GBS) based on clinical and electrophysiological criteria and a baseline Hughes Disability Score ≥ 3, were enrolled. Diagnostic criteria and eligibility were based on prior studies by Priyadarshini^8^ and Hughes^3^. Patients with previous GBS episodes, significant comorbidities, or contraindications to IVIG or corticosteroids were excluded. Independent assessors were used whenever feasible, but not consistently. No formal power calculation was performed; the study was exploratory in nature.

Randomization was performed using a computer-generated sequence, with allocation concealed by sealed opaque envelopes. The study was open-label, but whenever possible, clinical assessments were performed by independent evaluators to minimize bias.

The study was not prospectively registered and did not fully adhere to CONSORT guidelines, which represents a methodological limitation.

### Baseline evaluation

At admission, all patients underwent standardized laboratory testing, including complete blood count, renal and hepatic function tests, urinalysis, and cerebrospinal fluid (CSF) analysis. These investigations were intended to exclude alternative diagnoses and establish baseline clinical parameters, consistent with established protocols^11^.

### Treatment protocol

- Group A (*n* = 14): Received IVIG at 1 g/kg/day for two consecutive days, following the protocol recommended by Hughes^3^.
- Group B (*n* = 14): Received the same IVIG regimen in addition to intravenous methylprednisolone at 30 mg/kg/day (maximum 1000 mg/day) for five consecutive days, in line with Ma^7^.

All patients received supportive care, including close respiratory monitoring, hydration, and nutritional support, consistent with pediatric neurology guidelines^1,12^.

### Follow-up and outcome measures

Patients were assessed clinically at baseline, weekly during the first month, biweekly up to 12 weeks, and monthly up to 6 months. Outcomes included time to ambulation, Hughes Disability Score, neuropathic pain persistence, and adverse events.

- *Primary outcomes* time to unaided walking, change in Hughes Disability Score, and duration of hospitalization.
- *Secondary outcomes* time to initial clinical response, need for mechanical ventilation, persistence of neuropathic pain, relapse rate, and treatment-related adverse events.

This outcome framework was adapted from prior clinical trials in pediatric GBS^4,9^.

### Statistical analysis

Data analysis was performed using SPSS version 26. Normality was tested with the Shapiro–Wilk test. Continuous variables were expressed as mean ± standard deviation (SD) for parametric data or median [interquartile range, IQR] for non-parametric data. Comparisons between groups were made using the independent t-test or Mann–Whitney U test, as appropriate. Categorical variables were analyzed using Fisher’s exact test. Statistical significance was defined as *p* < 0.05, following standard recommendations^4^.

## Results

### Baseline characteristics

Twenty-eight children with Guillain–Barré syndrome (GBS) were enrolled and equally randomized into two groups: Group A (IVIG alone) and Group B (IVIG plus methylprednisolone). The mean age of participants was 6.5 ± 2.1 years, with no significant difference between groups (*p* = 0.726). Sex distribution was balanced (male-to-female ratio 7:7 in each group, *p* = 1.000). Clinical severity at baseline including autonomic dysfunction, cranial nerve palsy, bulbar involvement, and neuropathic pain was comparable between the two groups (*p* = 1.000 for all), confirming baseline homogeneity (Table 1).

**Table 1:** Comparison of demographic and baseline clinical features between the studied groups.

Variables		Total	Group A	Group B	P-value
		n=28 (%)	n=14 (%)	n=14 (%)	
Age (years)	Mean ± SD	6.5 ± 2.1	6.36 ± 2.15	6.64 ± 2.12	0.726
	Min–Max	4–11	4–11	4–11	
Gender	Male	14 (50.0)	7 (50.0)	7 (50.0)	1.000
	Female	14 (50.0)	7 (50.0)	7 (50.0)	
Autonomic dysfunction		7 (25.0)	3 (21.4)	4 (28.6)	1.000
Facial paralysis		7 (25.0)	3 (21.4)	4 (28.6)	1.000
Bulbar paralysis		7 (25.0)	3 (21.4)	4 (28.6)	1.000
Neuropathic pain		23 (82.1)	11 (78.6)	12 (85.7)	1.000
Duration of hospitalization (days)	Mean ± SD	10.9 ± 3.7	12.0 ± 4.08	9.86 ± 2.96	0.123
	Min–Max	6–22	8–22	6–16	
Duration from starting treatment until initial response (days)	Median [IQR]	4 [3–5.75]	4.5 [3–6]	3 [3–4]	0.064
	Min–Max	3–10	3–10	3–7	
Mechanical ventilation		10 (35.7)	6 (42.9)	4 (28.6)	0.695@@

Values present as number and percent were analyzed by Fisher exact test.

Values present as mean ± SD were analyzed by independent samples t-test.

Values present as median and IQR were analyzed by Mann–Whitny test.

### Hospitalization and early response

The mean duration of hospitalization was shorter in Group B (9.86 ± 2.96 days) compared with Group A (12.0 ± 4.08 days), although this difference was not statistically significant (*p* = 0.123).

The median time to initial clinical response was 3 (3–4) days in Group B, versus 4.5 (3–6) days in Group A, indicating a trend toward faster recovery in the steroid group (*p* = 0.064).

The requirement for mechanical ventilation was lower in Group B (28.6%) than in Group A (42.9%), but the difference did not reach statistical significance (*p* = 0.695).

### Functional outcomes

At admission, the median Hughes Disability Score was similar between groups (3.5 (3–4.25) in Group A, vs. 3 (3–4) in Group B, *p* = 0.501). By week 4, both groups demonstrated significant improvement, with median scores of 1.5 in Group A and 1 in Group B (*p* = 0.147). At 3 months, Group B showed significantly better functional recovery (median 0 (0–1)) compared with Group A (1 (0.75–1), *p* = 0.016). This difference was statistically significant but clinically modest, highlighting the need for cautious interpretation. By 6 months, both groups had achieved near-complete recovery (median 0, *p* = 0.366).

The time to unaided walking was numerically shorter in Group B (21 vs. 28 days), but this difference did not reach conventional statistical significance (*p* = 0.050).

Relapse occurred in 7 patients overall (25%), with no significant difference between groups (28.6% in Group A vs. 21.4% in Group B, *p* = 1.000).

Of particular clinical relevance, post-treatment neuropathic pain persisted in 57.1% of patients in Group A but was completely absent in Group B (*p* = 0.002), highlighting a significant benefit of adjunctive corticosteroid therapy. Pain resolution was complete in Group B, which may represent the most clinically meaningful finding. Functional and efficacy outcomes are summarized in Table 2.

**Table 2:** Comparison of treatment efficacy and functional outcomes between the studied groups.

Variables			Total	Group A	Group B	*P*-value
			*n* = 28 (%)	*n* = 14 (%)	*n* = 14 (%)	
Disability grade	On admission	Median [IQR]	3 [3–4]	3.5 [3–4.25]	3 [3–4]	0.501
		Min – Max	2–6	3–6	2–5	
	After 4 weeks	Median [IQR]	1 [1–2]	1.5 [1–2]	1 [0–2]	0.147
		Min – Max	0–4	1–3	0–4	
	After 3mo	Median [IQR]	1 [0–1]	1 [0.75–1]	0 [0–1]	0.016*
		Min – Max	0–2	0–2	0–2	
	After 6mo	Median [IQR]	0	0 [0–1]	0	0.366
		Min – Max	0–1	0–1	0–1	
Duration from starting treatment until recovery of unaided walking (days)		Median [IQR]	23.5 [20–33.75]	28 [20–45]	21 [18–24.25]	0.050*
		Min – Max	15–55	18–55	15–50	
Duration of ventilation (days)		Median [IQR]	0 [0–4]	0 [0–5]	0 [0–4]	0.389
		Min – Max	0–6	0–6	0–5	
No of relapses			7 (25.0)	4 (28.6)	3 (21.4)	1.000
Neuropathic pain after treatment			8 (28.6)	8 (57.1)	0 (0.0)	0.002*

Values present as number and percent were analyzed by Fisher exact test.

Values present as median and IQR were analyzed by Mann–Whitny test.

*: Significant.

### Adverse effects

Adverse events were generally mild and manageable. Weight gain and increased appetite were observed more frequently in Group B (50% each) compared with none in Group A (*p* = 0.006). Gastrointestinal upset occurred in two patients in Group B (14.3%), but this difference was not statistically significant (*p* = 0.481).

No cases of sleep disturbance, gastrointestinal hemorrhage, hyperglycemia, or hypertension were reported. Infections requiring antibiotics were slightly more frequent in Group B (21.4%) compared with Group A (14.3%), but without statistical significance (*p* = 1.000) (Table 3).

**Table 3:** Adverse effects of steroids among the studied groups during or within one week after stopping treatment.

Adverse effects	Total	Group A	Group B	*P*-value
	*n* = 28 (%)	*n* = 14 (%)	*n* = 14 (%)	
Weight gain	7 (25.0)	0 (0.0)	7 (50.0)	0.006*
GIT^#^ upset	2 (7.1)	0 (0.0)	2 (14.3)	0.481
Increased appetite	7 (25.0)	0 (0.0)	7 (50.0)	0.006*
Development of new infection treated with antibiotics	5 (17.9)	2 (14.3)	3 (21.4)	1.000
Sleep disturbances	0 (0.0)	0 (0.0)	0 (0.0)	–
Gastrointestinal hemorrhage	0 (0.0)	0 (0.0)	0 (0.0)	–
Development of hyperglycemia requiring insulin	0 (0.0)	0 (0.0)	0 (0.0)	–
Development of hypertension requiring drug treatment	0 (0.0)	0 (0.0)	0 (0.0)	–

^#^ GIT is Gastrointestinal Tract.

Values present as number and percent were analyzed by Fisher exact test.

*: Significant.

By the end of the six-month follow-up, both groups achieved near-complete functional recovery. However, the combination therapy group demonstrated faster early improvement and complete resolution of neuropathic pain, suggesting that adjunctive corticosteroid therapy may provide a meaningful clinical advantage in pediatric GBS, particularly in resource-limited contexts.

## Discussion

This prospective comparative study evaluated the impact of adding intravenous methylprednisolone to standard IVIG therapy in pediatric Guillain–Barré syndrome (GBS). Our findings suggest earlier recovery and complete resolution of neuropathic pain in the combination group. However, the difference in Hughes Disability Score at three months was modest and may not represent a major clinical benefit, without an increase in serious adverse events or relapse rates.

Previous studies have shown limited efficacy of corticosteroids when administered as monotherapy in GBS^5,6^. However, accumulating evidence suggests that corticosteroids may provide synergistic benefits when combined with IVIG. Surve^13^ reported improved early outcomes in adults receiving adjunctive corticosteroids, while recent pediatric studies from Asia demonstrated faster ambulation and better functional recovery with short-course methylprednisolone plus IVIG^7,8^. These findings are further supported by the Cochrane systematic review^14^, which highlights the potential role of corticosteroids in enhancing recovery when used as part of combination therapy. Our results are consistent with these observations, supporting the role of corticosteroids as an effective adjunctive therapy.

Of particular importance, neuropathic pain recognized as a frequent and disabling symptom in pediatric GBS was completely absent in the steroid-treated group. This finding is clinically significant, given that pain negatively influences rehabilitation, mobility, and quality of life^9^. The analgesic benefit observed may be explained by the immunomodulatory effects of corticosteroids, including suppression of pro-inflammatory cytokines and inhibition of complement-mediated axonal injury^10,15^.

By 6 months, both treatment groups demonstrated near-complete recovery, confirming the well-established long-term efficacy of IVIG^3,4^. However, the earlier trajectory of improvement in the combination group has potential clinical and health system implications. While faster recovery may reduce hospital stay and caregiver burden, our study did not formally assess cost-effectiveness or IVIG-sparing strategies, and such implications should be interpreted cautiously. These implications should therefore be interpreted with caution, particularly in resource-constrained healthcare systems such as Egypt, where IVIG is expensive and often limited in availability^11^.

Our findings are also consistent with regional data emphasizing the need for context-sensitive strategies. For example, Mohamed^16^ reported diagnostic delays and limited access to IVIG in Egyptian public hospitals, underscoring the potential role of corticosteroids as a widely available and affordable adjunct. Similarly, the landmark Egyptian study by El-Bayoumi^17^ demonstrated that plasma exchange was as effective as IVIG in ventilated children with GBS, providing an alternative in critical care. Taken together, these findings highlight the importance of developing accessible, cost-effective, and evidence-based treatment approaches tailored to resource-limited settings.

The relapse rate in our study (25%) was higher than that reported in larger pediatric cohorts^2,18^. Although not statistically different between groups, this observation warrants further investigation. Possible explanations include the relatively high baseline disability (median Hughes score ≥ 3 at admission) and methodological limitations such as small sample size and restricted follow-up duration. Future studies should include electrophysiological subtyping and longer follow-up periods to better characterize relapse dynamics.

Adverse effects were limited to expected steroid-related events, namely weight gain and increased appetite, which were mild and manageable. Importantly, no serious infectious, metabolic, or cardiovascular complications were observed, supporting the safety of short-course methylprednisolone in pediatric populations.

Among the evaluated outcomes, complete resolution of neuropathic pain in the steroid group represents the most clinically meaningful finding, given its impact on rehabilitation and quality of life.

In summary, our results suggest that adjunctive corticosteroid therapy provides meaningful early benefits in pediatric GBS, particularly with respect to pain control and functional recovery, without compromising safety. Therefore, our results should be considered exploratory and hypothesis-generating rather than definitive. Nonetheless, larger multicenter trials are needed to validate these results and to identify patient subgroups most likely to benefit from combination therapy.

## Conclusions

In conclusion, this exploratory study suggests that adjunctive short-course intravenous methylprednisolone may accelerate early recovery and eliminate neuropathic pain in pediatric GBS. These findings are hypothesis-generating and require validation in larger, multicenter, blinded trials before influencing clinical practice.

## Recommendations

We cautiously recommend further exploration of adjunctive methylprednisolone in pediatric GBS, particularly in resource-limited settings. However, our study does not support IVMP monotherapy or changes to current standard practice. Future multicenter trials with adequate sample size, standardized pain scales, and electrophysiological subtyping are essential to confirm these preliminary observations.

## Limitations

This study was limited by its small sample size, single-center design, and open-label methodology. Independent assessors were not consistently available, and pain assessment lacked standardized scales. Electrophysiological subtyping and antibody profiling were not performed. In addition, the study was not prospectively registered and did not fully adhere to CONSORT guidelines. These methodological constraints mean that our conclusions must be interpreted with caution.

## Data Availability

The datasets generated and analyzed during the current study are not publicly available due to institutional restrictions but can be obtained from the corresponding author upon reasonable request.
